# Hatching the Cleidoic Egg: The Role of Thyroid Hormones

**DOI:** 10.3389/fendo.2013.00063

**Published:** 2013-05-31

**Authors:** Bert De Groef, Sylvia V.H. Grommen, Veerle M. Darras

**Affiliations:** ^1^Department of Agricultural Sciences, La Trobe University, Melbourne, VIC, Australia; ^2^AgriBio, Centre for AgriBioscience, Melbourne, VIC, Australia; ^3^Department of Biology, Katholieke Universiteit Leuven, Leuven, Belgium

**Keywords:** altricial, bird, egg, embryo, hatching, precocial, reptile, thyroid hormone

## Abstract

A major life stage transition in birds and other oviparous sauropsids is the hatching of the cleidoic egg. Not unlike amphibian metamorphosis, hatching in these species can be regarded as a transition from a relatively well-protected “aqueous” environment to a more hazardous and terrestrial life outside the egg, a transition in which thyroid hormones (THs) (often in concert with glucocorticoids) play an important role. In precocial birds such as the chicken, the perihatch period is characterized by peak values of THs. THs are implicated in the control of muscle development, lung maturation and the switch from chorioallantoic to pulmonary respiration, yolk sac retraction, gut development and induction of hepatic genes to accommodate the change in dietary energy source, initiation of thermoregulation, and the final stages of brain maturation as well as early post-hatch imprinting behavior. There is evidence that, at least for some of these processes, THs may have similar roles in non-avian sauropsids. In altricial birds such as passerines on the other hand, THs do not rise significantly until well after hatching and peak values coincide with the development of endothermy. It is not known how hatching-associated processes are regulated by hormones in these animals or how this developmental mode evolved from TH-dependent precocial hatching.

Apart from being key metabolic hormones, thyroid hormones (THs) play an important role in development by controlling the growth and differentiation of almost every organ in the vertebrate body. The first clear evidence for the need of THs in vertebrate development came from frogs, where THs, in synergy with corticosteroids, control the transition from an aquatic larva to a terrestrial juvenile during metamorphosis. Superficially, hatching in birds and other oviparous sauropsids resembles anuran metamorphosis in that it marks a transition from an “aqueous” environment, to some extent protected against desiccation and predation by the eggshell, to a more exposed terrestrial life. The similarity is more striking when the endocrinology of hatching is considered, at least in precocial birds. In all precocial bird species studied to date, hatching is accompanied by and dependent on a rise in THs (and corticosteroids). We will first discuss these hormonal changes in precocial species and compare them to what is known in altricial birds and other oviparous sauropsids. Secondly, we will briefly review the role of THs in hatching and hatching-associated processes such as lung and gut maturation, the development of endothermy, and imprinting behavior.

## Thyroid Hormone Levels in the Perihatch Period

The hatching of the cleidoic egg is a major life stage transition in birds and other oviparous sauropsids, as well as in monotremes. Given its economic importance, the chicken (*Gallus gallus*) is arguably the most extensively researched species in this context. Chicken eggs are incubated for 21 days. On the 20th day of development (E20), the embryo pierces the membrane of the air chamber with its beak, a stage known as the internal pipping (IP) stage. After pulmonary breathing is established, the eggshell over the air chamber is cracked by the egg tooth present on the beak (external pipping, EP).

### Thyroid hormone levels in the perihatch period of precocial birds

**Figure 1:**
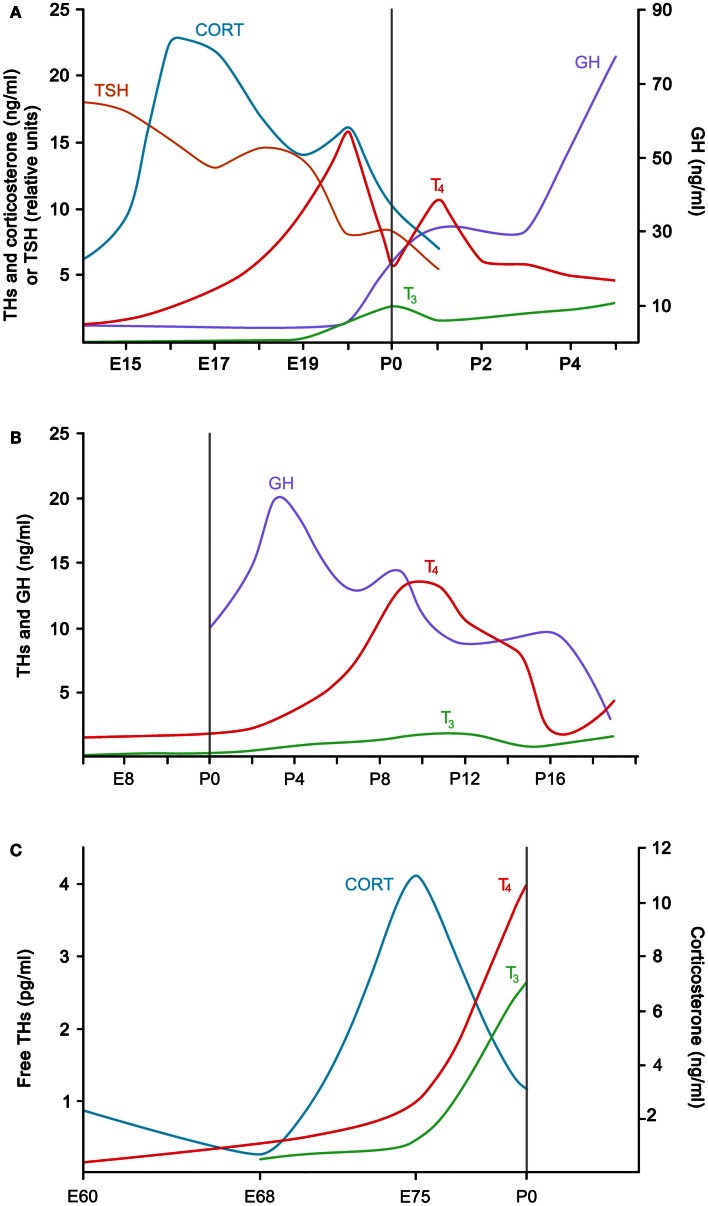
**Generalized ontogenic patterns of plasma hormones associated with hatching in (A) a precocial bird (chicken) (after Darras et al., 1992; Geris et al., 1998; and De Groef et al., 2006a), (B) an altricial bird (European starling) (after Schew et al., 1996) and (C) a non-avian sauropsid (saltwater crocodile) (after Shepherdley et al., 2002a)**. The vertical line in each graph refers to hatching. P, post-hatch day; CORT, corticosterone; E, embryonic day; GH, growth hormone, THs, thyroid hormones.

In the chicken, hatching is typically associated with peak values in circulating THs. In our 2006 study, for example, we measured values of around 13 pmol/ml thyroxine (T_4_) in the plasma during IP, compared to only 2 pmol/ml at E14 (De Groef et al., 2006a). Somewhat lagging behind to the rise in T_4_, plasma 3,5,3′-triiodothyronine (T_3_) concentrations showed a sharp ninefold increase between E19 (0.5 pmol/ml) and IP (4.5 pmol/ml). These patterns confirmed a number of earlier studies reporting the levels of circulating THs in the embryonic and hatching chicken (e.g., Thommes and Hylka, 1977; Decuypere et al., 1979; Darras et al., 1992; reviewed by Debonne et al., 2008) (Figure 1A).

The increased thyroid activity during the last week of chicken embryonic development is thought to be triggered predominantly by elevated hypothalamic stimulation of thyroid-stimulating hormone (TSH) secretion by the pituitary gland (De Groef et al., 2008). The effect of hypothalamic stimulation is amplified due to the concomitantly growing number of TSH-producing cells in the pituitary gland. In turn, the increasing levels of TSH are responsible for the gradual rise in circulating T_4_ levels during the last trimester of embryonic development. Intriguingly, which hypothalamic hormone drives the increasing TSH release is uncertain. In the chicken, two hypothalamic factors possess a potent TSH-releasing capacity: thyrotropin-releasing hormone (TRH) and corticotropin-releasing hormone (CRH) (reviewed by De Groef et al., 2006b). Hypothalamic TRH and CRH levels (mRNA and peptide) increase steadily toward hatching (Geris et al., 1999; Vandenborne et al., 2005; Lu et al., 2008; Ellestad et al., 2011) and the decreasing CRH peptide content of the median eminence toward E19 suggests elevated CRH secretion into the hypophyseal portal system (Vandenborne et al., 2005). TRH is at least in part involved in establishing T_4_ levels in the embryo in the second half of incubation: passive immunization with anti-TRH prevented the rise in plasma T_4_ levels which normally occurs between E10 and E13 (Thommes et al., 1988). The contribution of endogenous CRH, however, is still elusive.

Plasma T_3_ levels are very low during most of embryonic life, but increase abruptly around IP. The rise in T_3_ appears to be largely caused by a strong down-regulation of hepatic type 3 iodothyronine deiodinase (D3) expression, resulting in a more than 10-fold decrease in hepatic D3 activity from E16 to hatching (Darras et al., 1992; Van der Geyten et al., 2002). Owing to its inner ring deiodinating activity, D3 is a TH-inactivating enzyme, and a reduction of this enzyme causes T_3_ to accumulate instead of being broken down to inactive T_2_. The sudden change in hepatic D3 activity in the days leading to hatching is elicited by increased circulating levels of growth hormone and corticosterone (Figure 1A); both have been found to decrease the transcription of the *Dio3* gene encoding D3 in the chicken embryo (Van der Geyten et al., 1999, 2001). The observed increases in corticosterone, growth hormone, and T_3_ are all interrelated. Circulating corticosterone levels start to rise around E14 and act synergistically with THs on the differentiation of the growth hormone-producing cells in the pituitary gland (Jenkins and Porter, 2004; Liu and Porter, 2004; Porter, 2005). Corticosterone and growth hormone will then augment circulating T_3_ levels through their effect on D3.

Very similar patterns of circulating THs have been found in two species of quail (*Coturnix japonica* and *Colinus virginianus*), turkey (*Meleagris gallopavo*), and mallard duck (*Anas platyrhynchos*) (McNabb et al., 1981; Christensen et al., 1982; reviewed by McNabb, 2006). This suggests that, although only a limited number of species have been investigated, the ontogenic pattern of plasma THs found in chicken may be representative for precocial birds in general.

### Thyroid hormone levels in the perihatch period of altricial birds

**Figure 2:**
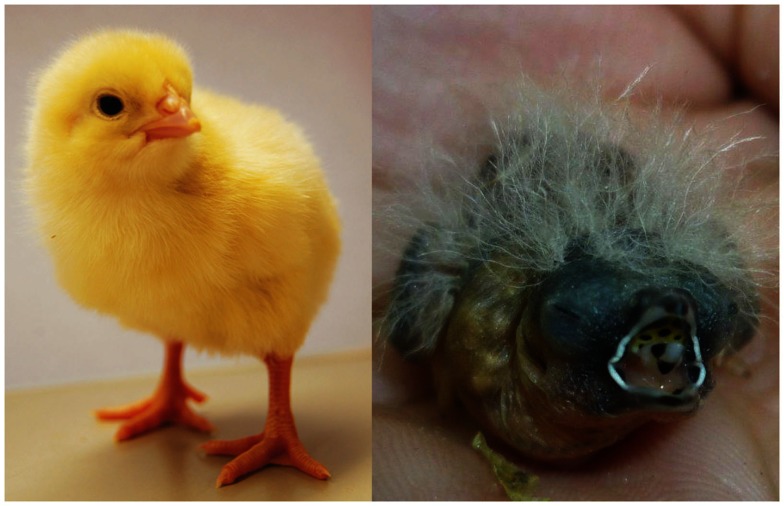
**Bird species with different developmental modes: the precocial chicken (*Gallus gallus*), 1 day old, and the altricial zebra finch (*Taeniopygia guttata*), 3 days old**. Photographs by Sylvia Grommen (chicken) and Nicola Khan (zebra finch).

Precocial birds like galliformes (fowl and relatives) and anseriformes (waterfowl and relatives) are at one end of a developmental spectrum characterized by differences in the chicks’ relative degree of maturation at hatching and the extent of parental care in the post-hatch period. Precocial birds are relatively well developed at hatching: in most galliformes and anseriformes, the eyes of the hatchlings are open and locomotion is good; the chicks are covered in down, capable of some thermoregulation, and seeking their own food (Starck and Ricklefs, 1998). This is in contrast to the altricial birds at the other end of the spectrum, like pigeons, passerines, and parrots. Fully altricial hatchlings hatch with closed eyes and ears, and exhibit little locomotor activity (Starck and Ricklefs, 1998). They hatch naked, show no thermoregulatory responses until some days or even weeks after hatching, and they are fully dependent on parental care (Figure 2). Although the distinction between precocial and altricial birds is largely based on morphological and behavioral traits, there are some marked differences in thyroid physiology as well: the perihatch peak of circulating THs is absent in ring doves (*Streptopelia risoria*) and in passerines like European starlings (*Sturnus vulgaris*), red-winged blackbirds (*Agelaius phoeniceus*), and great tits (*Parus major*) (Schew et al., 1996; Silverin and Rudas, 1996; Výboh et al., 1996; Olson et al., 1999; reviewed by McNabb, 2006), suggesting that this is the common pattern for altricial birds. Typically, circulating TH concentrations in these birds are very low during embryonic life, and then increase gradually to reach adult concentrations some days or weeks post-hatch (Figure 1B). Whereas the thyroid gland is fully developed at hatching in precocial birds, little thyroid development occurs in embryonic altricial birds (reviewed by McNabb, 2006). Yet other ontogenic patterns of plasma THs can be expected in species with intermediate developmental modes, such as semi-precocial and semi-altricial birds, or in birds with species-specific life stage transitions. Unfortunately, such studies are much underrepresented in the literature. A rare, if not the only example, is the study of the king penguin (*Aptenodytes patagonicus*) by Cherel et al. (2004). In semi-altricial king penguin chicks, plasma T_4_ followed the general altricial pattern post-hatch. However, plasma T_3_ had already reached adult values at hatching and did not change during the first growth phase (Cherel et al., 2004). THs later followed a pattern related to the unique life style of the bird (winter fast, spring mold, and departure to sea). This study highlights the need for more information on hormonal changes in different avian species with divergent patterns of development (Cherel et al., 2004).

### Thyroid hormone levels in the perihatch period of other oviparous sauropsids

In the saltwater crocodile (*Crocodylus porosus*), measurements of plasma free THs at three time points during the last quarter of the 80-day incubation showed a rise in circulating levels, the most significant increase occurring between day 75 of incubation and hatching (Shepherdley et al., 2002a) (Figure 1C). The concentrations of plasma T_3_ and T_4_ 1 day post-hatch were 22 times greater and 10 times greater, respectively, than those in plasma of juvenile (6-month-old) crocodiles. This study suggests that, at least in the saltwater crocodile, the ontogenic pattern of THs may be similar to that seen in precocial birds. Based on the histology of the thyroid follicles of embryos toward and during hatching, this may also be the case in other reptile species (Dimond, 1954; Miller, 1963). The ontogenic changes seen in deiodinase activities in the embryonic saltwater crocodile also resemble those of the chicken, most notably the marked decrease in hepatic D3 activity that could contribute to increasing plasma T_3_ levels toward hatching (Shepherdley et al., 2002b). In contrast to juvenile and adult crocodiles, the serum of crocodile embryos and 1-day-old hatchlings contains transthyretin, indicating that efficient TH delivery to the tissues is important during crocodile embryonic development (Richardson et al., 2005).

Very little is known about the circulating TH levels in the perihatch period of other non-avian species that lay cleidoic eggs, including the monotremes. While TH levels have been measured in juvenile and adult platypus (*Ornithorhynchus anatinus*) (Hulbert and Grant, 1983), no information is available on THs in embryos and hatchlings of monotremes.

## Thyroid Hormones and the Hatching Process of Precocial Birds

### Hatching

**Figure 3:**
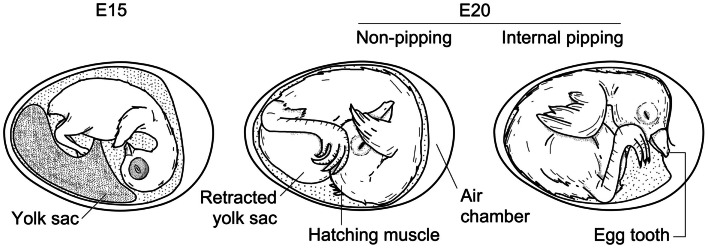
**Schematic comparison of the chicken embryo at days 15 and 20 of development, showing yolk sac retraction and internal pipping**. Drawing by Sylvia Grommen.

In the days leading to hatching, chicken embryos accumulate glycogen reserves in muscle and liver and show increased glycogenolysis to provide the energy needed for hatching and the initiation of thermoregulatory responses; chicks also initiate pulmonary respiration, retract their yolk sac, pip, and emerge (Figure 3). Grossowicz (1946) found that chicken embryos injected with the antithyroid drug thiourea between E7 and E17 were delayed in hatching up to as much as 10 days, and their yolk sacs were not retracted. This study was one of the first to highlight the role of THs in the hatching process. Since then, several studies using a variety of substances that interfere with TH homeostasis, including the environmental pollutant PCB 77 (Roelens et al., 2005), have been shown to affect the timing of hatching. Conversely, chicken embryos treated with THs (or TSH) hatch earlier (Balaban and Hill, 1971; Oppenheim, 1973) and premature yolk sac retraction and pipping were noted on E19 after a single injection of T_3_ or T_4_ on E16 (Decuypere et al., 1991). Similar observations have been made in one non-avian sauropsid species, namely in snapping turtle embryos (Dimond, 1954).

Thyroid hormones are involved in many different aspects of or processes associated with hatching (see further). They may also affect the hatching process more directly by coordinating the growth and development of the musculus complexus, also known as the “hatching muscle” (see Figure 3). Its primary function is to help the embryo pierce the egg membranes and shell during hatching (Smail, 1964). The hatching muscle reaches its maximum size at about E20 – mainly because it becomes filled with interstitial fluid – and rapidly becomes smaller after hatching (Pohlman, 1919). Treatment with thiourea delays or prevents the hatching muscle’s development (Brandstetter et al., 1962). Therefore, hypothyroidism may inhibit hatching because the hatching muscle is not fully developed and the animals are too weak to pip and emerge. In a recent study by Akhlaghi et al. (2012), for example, none of the eggs of broiler breeders made hypothyroid by 6-*N*-propyl-2-thiouracil (PTU) treatment hatched, even though the embryos were viable. Both plasma T_3_ and T_4_ levels were found to be significantly lower in the offspring of hypothyroid hens at E18 and IP compared to control embryos.

### Hatching as a transition in respiration

Hatching is the transition from an “aqueous” to a terrestrial environment that requires a switch from chorioallantoic to pulmonary respiration. The onset of lung respiration is accelerated by T_4_ and retarded by thiourea, respectively (Wittmann et al., 1983, 1984). It has been speculated that the T_4_-accelerated initiation of lung respiration causes relative hypoxia in the air chamber, which in turn is a stimulus for increased activity and hatching (Wittmann et al., 1983). Consequently, when T_4_ treatment accelerates lung respiration, hatching will also occur earlier. Embryos treated with thiourea did not initiate lung ventilation, and the associated intensified body movements, increased metabolism, and elevated oxygen consumption were not observed (Wittmann et al., 1984). These experiments suggest a pivotal role for THs in lung maturation and hence hatching. A successful transition of chorioallantoic to pulmonary respiration requires both a reduction of alveolar surface tension and an increased blood supply to the lungs. Whereas surfactant maturation in mammals is controlled predominantly by glucocorticoids and THs working synergistically, the precise role of THs in lung maturation in sauropsids may be more variable. In sauropsid embryos, content and synthesis of pulmonary surfactant phospholipids increase prior to the onset of breathing, i.e., prior to pipping in the chicken (Hylka and Doneen, 1982; Sullivan and Orgeig, 2001), and at hatching in non-avian sauropsids (Sullivan et al., 2001, 2002a). THs were shown to regulate surfactant phospholipid secretion by pulmonary type II cells isolated from the green sea turtle (*Chelonia mydas*) and saltwater crocodile; in the bearded dragon (*Pogona vitticeps*) T_3_ was only tested in combination with dexamethasone, a synthetic glucocorticoid (Sullivan et al., 2001, 2002a,b). T_3_ alone (without addition of dexamethasone) was also capable of increasing the production and saturation level of surfactant phospholipids in the embryonic saltwater crocodile *in vivo* (Sullivan et al., 2002b). In the chicken embryo, however, corticosterone triggered surfactant phospholipid synthesis, whereas THs, alone or in addition to glucocorticoids, generally had no effect on surfactant production (Hylka and Doneen, 1983; Blacker et al., 2004). Similarly, hypoxia from E10 onward was found to accelerate surfactant maturation and hatching, but elicited only a rise in circulating corticosterone and not T_3_ levels (Blacker et al., 2004). However, a role for THs in chicken lung maturation cannot be excluded based on these experiments alone. Sharply increased TH receptor β (TRβ) mRNA expression was evident in lung tissue on E19 compared with E16 where it was almost undetectable (Forrest et al., 1990). This suggests that lung maturation occurs during a TH-sensitive period. It is possible that THs mainly act to increase the sensitivity of the lung tissue to glucocorticoids, and/or that the effects of THs were already maximal at the ages tested so that further stimulation with exogenous THs did not result in an additional effect. In addition, Blacker et al. (2004) found evidence that THs may have a role in increasing the saturation of phospholipids early in surfactant development (E16), probably via enhanced surfactant synthesis rather than secretion.

Thyroid hormones also seem to affect the blood flow in the maturing chicken lungs. Pulmonary vascular resistance is reduced during the transition from chorioallantoic to pulmonary respiration, so that blood flows preferentially to the lungs. This process is believed to be controlled by the kallikrein–kinin system (reviewed by Decuypere et al., 1991). In birds, the formation of vasoactive ornithokinin is catalyzed by the enzyme ornithokallikrein, whereas angiotensin-converting enzyme (ACE) is responsible for the degradation of the kinin. In the last few days of chicken embryonic development, the activity of both enzymes increases. After IP, the activity of ornithokallikrein continues to increase, while the activity of ACE does not (Wittmann et al., 1987). Thiourea treatment at E17 prevented the increase in ornithokallikrein activity and the attenuation of the increase in ACE activity (Wittmann et al., 1987), thus pointing to a role for THs in balancing pulmonary kinin production. It should be noted, however, that the involvement of the kallikrein–kinin system in embryonic lung maturation in sauropsids is assumed by analogy to mammals and, to our knowledge, has never been proven experimentally. Likewise, direct effects of THs on kallikrein and ACE enzyme activities and/or gene expression, unlike in mammals, have not been investigated in sauropsid species.

### Hatching as a transition in diet

Hatching generally marks the transition from a yolk-based diet (consisting mainly of lipids) to a solid feed diet (containing mainly carbohydrates and proteins). Associated with this process are the maturation of the gastrointestinal tract and the retraction of the yolk sac. Birds hatch with an immature gastrointestinal tract with the yolk sac still attached. In chicken, the yolk sac is progressively retracted into the abdominal cavity during the last 2 days of embryonic development until about 14 h before hatching (El-Ibiary et al., 1966). As mentioned above, manipulation of the TH levels during late embryonic development affects retraction of the yolk sac. A remarkable elevation of TRβ mRNA levels was found in the yolk sac, with expression increasing gradually from the earliest detectable stage (E7) until E19 where levels were elevated 30-fold (Forrest et al., 1990). Even though yolk sac retraction has been unequivocally attributed to the effects of THs (Wishart et al., 1977), the precise mechanisms remain unknown.

Toward the end of incubation, the gastrointestinal tract of the chicken embryo changes dramatically, both morphologically and functionally, but the final maturation of the gut occurs post-hatch. The weight of the intestine as a proportion of embryonic weight increases 3.5 times from E17 to hatching and the small intestine increases in weight faster than the body weight during the first week post-hatch (reviewed by Sklan, 2001). The post-hatch growth period is characterized by an increasing number of enterocytes, and development of the villi and crypts. Consequently, the ability of the intestine to digest and absorb nutrients increases post-hatch. Brush border and pancreatic enzyme expression, as well as the expression of intestinal transporters also change to accommodate the new diet (Uni et al., 2003; Uni and Ferket, 2004; and references therein). There are indications that THs play a role in the functional maturation of the intestine in the chicken embryo. Administration of thiourea prevented differentiation of the intestinal epithelium (Moog, 1961). This finding was further supported by a series of *in vitro* experiments in which duodena from E12 to E18 chicken embryos were cultured in medium. T_4_ was found to greatly increase the number of goblet cells in explant cultures (Black and Moog, 1977) and to enhance the differentiation rate of the absorptive cells, particularly stimulating the outgrowth of microvilli (Black, 1978). Addition of T_4_ to the medium also resulted in a large increase in alkaline phosphatase activity and release of maltase activity from the intestinal tissue (Black and Moog, 1978) and caused alterations in the carbohydrate metabolism of the intestine (Black, 1988). Glycogen accumulation in the epithelial cells was reduced and glucose oxidation and utilization were increased by T_4_ (Black, 1988). In live embryos, T_4_ treatment resulted in precocious development of duodenal morphology, alkaline phosphatase activity, and sugar uptake (Mallon and Betz, 1982). In general, the available data suggest the involvement of THs – often in concert with glucocorticoids – in normal intestinal development, at least in the late embryonic stages.

The switch in energy sources that a hatchling goes through is accompanied by substantial changes in metabolism. Expression of lipogenic enzymes in the liver enables the newly hatched chick to convert dietary carbohydrate into fat stores (Speake et al., 1998). A differential gene expression study by Cogburn et al. (2003) revealed a number of hepatic genes involved in lipogenesis to be upregulated in chicken hatchlings compared to E16–E20 embryos. At least some of the differentially expressed genes are known to be regulated by THs, most notably *THRS* (TH-responsive Spot 14), *C/EBP*α (CCAAT/enhancer-binding protein α), and *PPAR*γ (peroxisome proliferator-activated receptor γ), all T_3_-regulated transcription factors that drive a number of enzymes involved in lipogenesis (Cogburn et al., 2003, 2007; Wang et al., 2007).

Altricial birds generally have relatively larger intestines at hatching than precocial birds (reviewed by Starck, 1998). However, only very few studies have addressed the morphological and physiological development of the digestive system in altricial birds. Caviedes-Vidal and Karasov (2001) found that in nestling house sparrows (*Passer domesticus*) digestive organs reach their highest mass by post-hatch day 6 (stomach, intestine) or 9 (liver, pancreas), showing a more than 10-fold increase compared to hatch. Likewise, the levels of some digestive enzymes, e.g., sucrase and maltase in mid-intestine, increased more than 10-fold in the first 12 days post-hatch, and pancreatic amylase activity increased 100 times during this period (Caviedes-Vidal and Karasov, 2001). In domestic pigeons (*Columba livia*), intestinal morphology, and mucosal and pancreatic enzyme activities also change rapidly during the first 14 days post-hatch (Dong et al., 2012). However, none of these studies considered embryonic development of the digestive system, or the involvement of THs in pre- or post-hatch gut development. Given the extensive variation in diets in altricial nestlings (e.g., crop milk in pigeons, insects in insectivorous species etc.), it is likely that the functional and morphological maturation of the digestive tract is equally varied between species. To what extent this variation is also reflected in the involvement of THs is unknown.

### Hatching as a transition in thermoregulation

The molecular mechanisms underlying endothermy in birds have been the subject of much controversy. While the transcriptional coactivator PGC1α, a major regulator of metabolism, and adenine nucleotide translocase, the principal uncoupling protein in birds, were found to be TH-dependent in the chicken, methimazole (MMI)-induced hypothyroidism did not affect oxidative capacity and uncoupling of mitochondrial respiration during the development of endothermy (Walter and Seebacher, 2009). This study suggests that THs do not act as overall controllers of capacity, uncoupling, and basal energy demand during the development of endothermy in the chicken. Nevertheless, when circulating TH patterns are compared in precocial versus altricial birds, they mostly seem to parallel the development of endothermy and thermoregulation. THs, especially T_3_, are thermogenic in the post-hatch chicken (Freeman, 1970; Decuypere et al., 1981; Klandorf et al., 1981). In the precocial chicken, thermoregulatory mechanisms become functional in the late embryonic stage (Freeman, 1964; Tazawa et al., 1988). Young embryos are essentially ectothermic, but weak metabolic responses to cooling are sometimes seen in older embryos, unless they were treated with thiourea (Tazawa et al., 1989). E16.5 embryos can respond to cooling by increasing plasma T_4_ levels (Thommes et al., 1988). After EP, the response to cooling is stronger (Tazawa et al., 1988). Likewise, late prenatal ducks, but not altricial pigeon hatchlings or semi-altricial brown noddy (*Anous stolidus*) embryos, show evidence of thermoregulatory responses (Matsunaga et al., 1989; Kuroda et al., 1990). In altricial species, the development of endothermy is delayed until some weeks after hatching, and so is the maturation of the thyroidal axis. The development of the thyroidal axis and its possible relationship with the initiation of thermogenesis has been extensively reviewed elsewhere (McNabb and Olson, 1996; McNabb, 2006).

## Thyroid Hormones and Post-Hatch Imprinting Behavior

The development of thermogenesis not only requires maturation of thermogenic mechanisms, but also a sufficiently developed nervous system coordinating the thermosensors and controllers. The role of THs in late brain maturation is well established. TRα and TRβ are expressed in the brain (both in similar areas) during embryonic development (Forrest et al., 1991). Hypothyroidism induced a week before hatching may therefore have implications on TH-dependent gene expression, affecting processes such as neuronal proliferation and migration (Bouvet et al., 1987; Verhoelst et al., 2004). Besides from playing an important role during late embryonic brain development, THs have also been shown recently to impact early post-hatch processes such as learning behavior. Kagami et al. (2010) found that pre-hatch hypothyroidism induced by the injection of either MMI or PTU in the egg at E14 resulted in altered imprinting behavior in newly hatched chickens. Filial imprinting is the learning process that newly hatched chicks and ducklings undergo when they are exposed to the parent birds, normally the first moving object they see, and start to follow them around (Lorenz, 1937). The MMI- and PTU-treated chicks demonstrated a significantly lower preference for the imprinting object compared to the control group when trained and tested respectively the first and second day after hatching, which is indicative of an impaired learning ability and a role for THs therein. Recently, Yamaguchi et al. (2012) have demonstrated that THs are essential for the visual imprinting process in the chicken, and that the effect of T_3_ on imprinting is likely mediated via non-genomic action. T_3_ was also found to be involved in regulating the timing of the sensitive period. Normally this susceptible period only exists for the first 3 days after hatching, but the exogenous administration of T_3_ before training on post-hatch days 4 and 6 (but not on day 8) was able to “re-open” the previously closed sensitive window. It was suggested that, since the injection of T_3_ was effective at concentrations similar to those observed at post-hatch day 1, the sensitive period of imprinting might be regulated by the perihatch change in THs levels (Yamaguchi et al., 2012).

It is unknown whether THs are also involved in filial imprinting and in determining the start of the sensitive period in altricial birds. In contrast to precocial chicks, altricial young have their eyes closed when hatched. The timing of when the nestlings open their eyes varies between species and is linked to the degree of altriciality. Zebra finch (*Taeniopygia guttata*) chicks, for example, have fully open eyes around day 10 post-hatch and start responding to visual stimulation at that time (Bischof and Lassek, 1985), while red-winged blackbird nestlings have their eyes already fully open around day 7–8 (Holcomb and Twiest, 1971). The timing of the sensitive period of imprinting, whether or not regulated by THs, will likely vary depending on the species and differences in developmental mode. Junco (1988) examined filial imprinting in Eurasian blackbird (*Turdus merula*) nestlings using a training model that elicited begging behavior. The optimal period of imprinting was found to be at 11 days post-hatch. At days 10 and 12 there was no specific preference for the training object. This suggests that there is an increased sensitivity for imprinting at a particular time point, though this sensitive window seems to be shorter than that observed in the chicken. Whether or not this correlates with high levels of THs in plasma or brain still needs to be determined, but considering the post-hatch TH profiles observed in other altricial birds, this is likely to be the case.

## Conclusion

**Figure 4:**
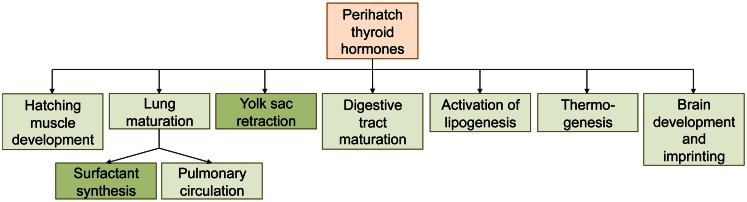
**Overview of the roles of thyroid hormones in processes associated with hatching in precocial birds (chicken)**. Processes in darker boxes have been shown to be thyroid hormone-regulated in non-avian sauropsids as well.

Thyroid hormones are intimately linked with the hatching process in precocial birds, and perhaps also non-avian sauropsids (summarized in Figure 4). This raises questions about the endocrine control of hatching in altricial birds. Whereas hatching is delayed or even inhibited when the circulating TH levels in precocial birds are experimentally prevented to rise, altricial birds naturally hatch in the absence of high TH concentrations. Whether hatching and the associated processes such as yolk sac retraction, gut maturation, and the initiation of pulmonary respiration are therefore completely independent of TH control in altricial birds is not known; to our knowledge, the effect of antithyroid drugs on these processes has not been investigated in altricial birds. It has been suggested that the peak in THs during late embryogenesis in precocial birds is the result of the initiation of thermoregulation, representing a response to the cooling that occurs during hatching (Freeman, 1970). This is seemingly supported by the association of TH peak values and the onset of thermoregulation during nestling in altricial birds. However, this hypothesis is based on temporal correlations only and is hard to reconcile with the (albeit very limited) evidence that in ectothermic reptiles too, hatching seems to be associated with increased thyroid activity. Given that the avian taxa showing TH-regulated precocial hatching (i.e., Galloanserae) are more primitive than those having altricial development, and that almost all extant non-avian sauropsids are precocial, it can be assumed that the altricial developmental mode evolved later. The question then is whether (and if so, how) altricial birds “detached” their hatching processes from TH control, or whether the strong TH dependence of hatching processes in precocial birds is a derived trait (i.e., whether THs were co-opted to regulate hatching-associated processes). This question identifies two major gaps in our current knowledge of the endocrine control of the hatching of the cleidoic egg: (1) to understand how the endocrine control of hatching evolved, more experimental data in non-avian sauropsids and altricial birds are needed. Whereas precocial birds may be more interesting from an economic point of view, studies on non-avian sauropsids and altricial birds may yield useful information for wildlife conservation such as *ex situ* breeding programs. Semi-altricial, semi-precocial, and precocial species other than Galloanserae should be included as well. Of particular interest are species where precocity evolved secondarily from an altricial ancestor, such as in Gruiformes (cranes, rails, and relatives) (Ricklefs and Starck, 1998). It is not known whether hatching in these secondary precocial species is also tightly controlled by THs. (2) While it is clear that THs play a pivotal role in hatching in precocial birds, the molecular mechanisms controlled by THs are largely unknown and little progress has been made in the last 20 years. In many cases, the involvement of THs has been determined based on the effects caused by treatment with endogenous THs or with antithyroid drugs. Differential gene expression studies are now starting to unravel the developmental pathways controlled by THs at the molecular level.

## Conflict of Interest Statement

The authors declare that the research was conducted in the absence of any commercial or financial relationships that could be construed as a potential conflict of interest.
